# Subclinical hypothyroidism and the risk of cancer incidence and cancer mortality: a systematic review

**DOI:** 10.1186/s12902-020-00566-9

**Published:** 2020-06-09

**Authors:** Juan Gómez-Izquierdo, Kristian B. Filion, Jean-Franҫois Boivin, Laurent Azoulay, Michael Pollak, Oriana Hoi Yun Yu

**Affiliations:** 1 Department of Family Medicine, McGill University, Montreal, Quebec Canada; 2 Division of Clinical Epidemiology, Department of Medicine, McGill University, Montreal, Quebec Canada; 3 Center for Clinical Epidemiology, Lady Davis Institute, Jewish General Hospital, 3755 Côte Sainte-Catherine, H-425, Montreal, Quebec H3T 1E2 Canada; 4 Department of Epidemiology, Biostatistics, and Occupational Health, McGill University, Montreal, Quebec Canada; 5 Gerald Bronfman Department of Oncology, McGill University, Montreal, Quebec Canada; 6 Department of Oncology, McGill University, Montreal, Quebec Canada; 7 Segal and Goodman Cancer Centres of McGill University, Montreal, Quebec Canada; 8 Division of Endocrinology, Department of Medicine, Jewish General Hospital, Montreal, Quebec Canada

**Keywords:** Subclinical hypothyroidism, Cancer, Systematic review, Mortality

## Abstract

**Background:**

Thyroid hormone has been shown to be involved in carcinogenesis via its effects on cell proliferation pathways. The objective of this study is to determine the association between subclinical hypothyroidism (SCH) and the risk of incident cancer and cancer mortality via systematic review.

**Methods:**

A systematic search was performed on Medline and Pubmed to identify relevant studies. Randomized controlled trials, and observational studies assessing SCH or its treatment and the risk of incident cancer or cancer mortality were identified.

**Results:**

A total of 7 cohort and 2 case-control studies met our inclusion criteria. In general, these studies were of medium to good quality. Overall, studies revealed no association between SCH and breast and prostate cancer. One study found that untreated SCH may be associated with an increased risk of colorectal cancer (adjusted odds ratio [OR]: 1.16; 95% confidence interval [CI]: 1.08–1.24). One study showed an increased risk in thyroid cancer incidence (adjusted OR: 3.38; 95% CI: 2.05–5.59) associated with elevation of a thyroid stimulating hormone (TSH) of > 1.64mIU/L. Two studies found an increase in cancer mortality among patients with SCH compared to euthyroid individuals; in contrast one study found no association between subclinical hypothyroidism and cancer mortality among aging men.

**Conclusion:**

The number of studies examining thyroid dysfunction and cancer risk and mortality is limited. Future studies assessing the association between thyroid dysfunction and cancer risk and mortality are needed, which will further address the need to treat subclinical hypothyroidism.

## Background

The relationship between thyroid function and cancer has been a subject of debate for more than 200 years, with studies showing conflicting results [1, 2]. In vitro studies have shown that thyroid hormones not only regulate body metabolism but they also play an important role in cell proliferation and differentiation in normal tissues [3]. Thyroid hormones consist of thyroxine (T4) and triiodothyronine (T3), which is the active form of the thyroid hormone. Both enter cells via transporter proteins whereby T4 becomes converted to T3 by deiodinases. T3 subsequently binds to thyroid hormone receptors, which then forms heterodimers with the retinoid X receptor (RXR) to induce transcription of a number of target genes with thyroid response elements [3].

Subclinical hypothyroidism (SCH) is a form of thyroid dysfunction that is highly prevalent, with a reported prevalence of 5 to 10% of the population worldwide [4]. SCH is defined by elevated thyroid stimulating hormone (TSH) levels with normal T4 levels. Given that the T4 level is within normal range, patients with SCH usually do not experience hypothyroid symptoms. However, given that studies have shown an increased risk of heart failure associated with SCH whereby the TSH levels are > 10 mIU/L [5], it is recommended that patients with SCH and this extent of TSH elevation receive levothyroxine replacement [6]. Currently, there is no consensus as to whether patients with SCH with a TSH level of < 10mIU/L should be treated with levothyroxine as there is uncertainty regarding the clinical benefits of therapy in these cases. According to some studies, less than 50% of patients with SCH receive hormone replacement [7].

Given that thyroid hormone has been shown to play a role in cancer pathogenesis, further studies assessing the association between thyroid dysfunction and its effects on carcinogenesis are needed. This is particularly true for SCH, where the clinical benefits of levothyroxine treatment are unclear. The objective of this study is therefore to determine the influence of thyroid dysfunction, namely SCH, on cancer incidence and cancer mortality via systematic review of the evidence available to date.

## Methods

### Data source and searches

A systematic search was performed on Ovid MEDLINE from the date of its inception until November 13th, 2017, combining words related to thyroid and cancer (MeSH and non- MeSH terms) to identify studies of thyroid dysfunction and incident cancer and those examining thyroid dysfunction and mortality in cancer patients (Appendix). A second search was performed using Pubmed from the date of its inception until March 12, 2020 (Appendix). No language limitations were used. In addition, we hand-searched the references of included studies to identify additional relevant studies that were not identified in our electronic search.

### Study selection

Inclusion criteria comprised of randomized clinical trials assessing treatment of SCH with levothyroxine treatment, cohort and case-control studies reporting SCH, where the thyroid dysfunction chronologically preceded the cancer incidence or mortality by at least a year to reduce the possibility of including studies with reverse causation, meaning that the cancer or its treatment induced the thyroid dysfunction and not the opposite [8]. Articles where the SCH was primary (not secondary to a medical treatment or procedure) were included. Articles reporting iatrogenic hypothyroidism, thyroid dysfunction during pregnancy or were medication induced were not included since the effect of these causes of SCH on cancer might have a different pathophysiology considering that SCH is commonly induced by an autoimmune mechanism [9, 10]. Exclusion criteria were studies using outdated methods to screen or diagnose thyroid dysfunction or cancer, suspected to have a high risk of bias (i.e. recall) according to the study methods, and studies in which the reference and comparison groups have cancer. Studies were screened by two investigators (J.G. and O.Y.) and discrepancies were resolved by a third investigator (K.B.F.).

### Data extraction and quality assessment

Data were extracted using a pilot-tested form that included author, year of publication, study design, study period, sample size (overall and by group), type of population, age of the patients, source of the data or database used for the study, follow-up duration, objective of the study, if anti-thyroperoxidase antibodies (TPOAb) had been measured, effect measure, results (adjusted rates, survival times, and mortality rates), and conclusions.

Quality assessment was performed using the Cochrane tool to assess risk of bias in cohort studies [11] and the Newcastle-Ottawa quality assessment scale for case control studies [12]. Data extraction and quality analyses were completed independently by two authors (J.G. and O.Y.). Disagreements were resolved by discussion with a third independent reviewer (K.B.F.).

Meta-analysis was not possible due to the high heterogeneity in study designs, effect measures reported, and outcomes (cancer incidence, mortality, survival time). All the methods followed the PRISMA guidelines for systematic reviews of the literature [13, 14].

## Results

### Study selection

**Fig. 1:**
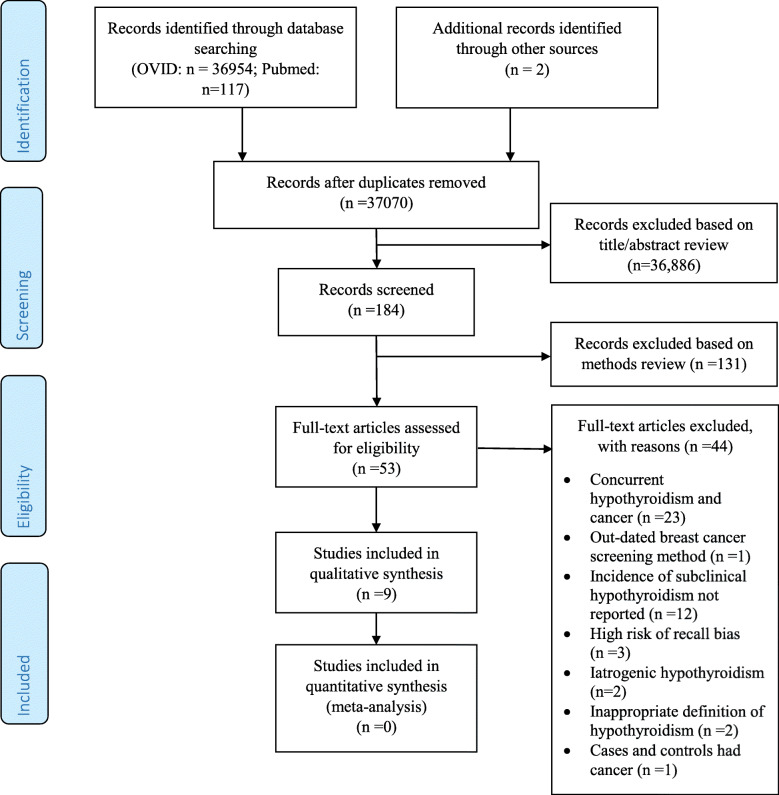
PRISMA Flow Diagram showing the selection of studies assessing the association between subclinical hypothyroidism and cancer incident risk and cancer mortality. The flow diagram template was adapted from the 2009 PRISMA statement [14]

A total of 37,073 records were identified (Fig. 1); 36,954 were retrieved from Ovid MEDLINE, 117 were retrieved from Pubmed and 2 were retrieved from other sources [15, 16] (cited in other articles obtained via our search). 36,886 articles were found non-relevant after screening the titles and abstracts. One hundred and eighty-four articles were screened via further assessment of the methods described and subsequently 53 articles were thoroughly reviewed for eligibility. Forty-four articles were excluded for the following reasons: 23 studies identified concurrent thyroid dysfunction and cancer (reverse causation cannot be excluded) [17–38], 1 study used an outdated method to screen for breast cancer (i.e. thermography) [39], 12 studies did not report SCH [40–51], 3 studies have potential high risk of recall bias (no specific tool was applied to assess for risk of recall bias at this point; it was identified by the assessor after reading the methods of the article) [52–54], 2 studies included iatrogenic hypothyroidism [55, 56], 2 studies had an inappropriate definition of hypothyroidism (both defined hypothyroidism based on use of thyroid hormone replacement without reporting levels of TSH or T4) [57, 58], and 1 study involved cases and controls with a history of cancer at baseline [59]. A total of 9 studies were included in our systematic review.

### Study characteristics

The 9 included studies comprised of 2 case-control studies [7, 60], 3 retrospective cohort studies [16, 61, 62], and 4 prospective cohort studies [8, 15, 63, 64] (Tables 1 and 2). There were no randomized controlled trials identified that studied the effects of treatment of SCH on the risk of cancer or cancer mortality. One included study assessed the effect of SCH on colorectal cancer [7], one study on breast cancer [8], one study on prostate cancer [60], one on thyroid cancer [61] and one on hepatocellular carcinoma [62]. One study analyzed overall cancer incidence [15], and three studies focused on overall cancer mortality [16, 63, 64]. Two of these studies assessed cancer mortality as a secondary outcome [16, 64].

**Table 1:** Study characteristics of comparative studies evaluating subclinical hypothyroidism and cancer risk and mortality

Study	Study design	n	Data origin	Study period	Population	Definition of SCH
Kuijpens 2005 [8]	Prospective cohort	2738	The Eindhoven Cancer Registry	1994–2003	All women between 47 and 54 years old living in the city of Eindhoven were invited to participate in the Eindhoven perimenopausal osteoporosis study. All women who did not have breast cancer in 1994 were followed	TSH > 6mIU/L and T4 within normal range (8 to 26 pmol/L)
Hellevik 2009 [15]	Prospective cohort	29,691	Nord-Trøndelag Health Study	1995–2005	Participants completed questionnaire with thyroid function tests drawn and were followed for cancer incidence, defined using the Cancer Registry of Norway	TSH > 3.5mIU/L
Razvi 2012 [16]	Retrospective cohort	4735	United Kingdom General Practitioner Research Database	2011–2009	Patients with incident subclinical hypothyroidism followed for ischemic heart disease and all-cause mortality (cancer mortality also assessed).	TSH 5.01 to 10mIU/L
Mondul 2012 [60]	Case-control	1201	Alpha-Tocophenol, Beta-Carotene Cancer Prevention Study	1985–1993	Prostate cancer patients diagnosed 3 years after baseline matched with up to 2 controls	TSH > 3mIU/ L; T4 < 4.6 μg/dL
Waring 2012 [64]	Prospective study	1337	Osteoporotic Fractures in Men Study (MrOs) Cohort	2000–2011	Men ≥65 years of age in six clinical centers in the United States	TSH above upper limit of normal and < 10 mU/L
Fighera 2015 [61]	Retrospective cohort	622	Federal University of Parana	1999–2008	Patients with subclinical hypothyroidism with thyroid nodules that had thyroidectomy versus fine needle aspiration biopsy	TSH was assessed as a continuous variable
Boursi 2015 [7]	Nested case-control	103,044	The Health Improvement Network (THIN)	1995–2013	Case patients identified in THIN with colorectal cancer matched to up to 4 eligible control patients	TSH > 4 mg/dL
Tseng 2015 [63]	Prospective cohort	115,746	Taiwan	1998–2008	Patients with no known thyroid disorders on medication treatment that had a health examination in one of 4 private nationwide MJ Health Screening Centers in Taiwan, followed for cancer mortality	TSH 5 to 19.96 mIU/L
Pinter 2017 [62]	Retrospective cohort	667	Medical University of Vienna	1992–2013	Patients diagnosed with hepatocellular carcinoma with thyroid function tests, followed for overall survival	Free T4 ≤ 1.66 ng/dL

Abbreviations: *n* number of individuals, *SCH* subclinical hypothyroidism, *TSH* thyroid stimulating hormone, *T4* thyroxine

**Table 2:** Effect estimates of cancer risk and mortality in studies comparing patients with untreated to treated subclinical hypothyroidism or euthyroidism

Study	Treated SCH/ euthyroid (n)	Untreated SCH (n)	Effect measure	Point estimate	95% CI	TPOAb	Finding summary
Kuijpens 2005 [8]	NA	NA	OR	1.9	0.8–4.9	More prevalent in women with previous or current diagnosis of breast cancer.	There was no association between subclinical hypothyroidism and the risk of breast cancer. However, women with a history of breast cancer were more likely to have anti-TPO antibodies.
Hellevik 2009 [15]	12,389	2149	HR	0.96	0.82–1.12	NA	There was no association between the risk of overall cancer incidence and TSH level of > 3.5mIU/L.
Mondul 2012 [60]	800 prostate cancer patients	401 controls	OR	0.71	0.47–1.06	NA	Men with elevated TSH levels were associated with a decreased risk of prostate cancer.
Razvi 2012 [16]	1634 (age 40–70); 819 (age > 70)	1459(age 40–70); 823 (age > 70)	HR	0.59 (age 40–70) 0.51 (age > 70)	0.21–0.99 (age 40–70) 0.24–1.09 (age > 70)	NA	Treatment of SCH was associated with a decreased risk of cancer mortality among adults age 40 to70 years.
Waring 2012 [64]	1248 (men age ≥ 65)	89 (men age ≥ 65)	RH	0.88	0.44–1.74	NA	Subclinical hypothyroidism was not associated with cancer mortality among men ≥65 years of age.
Boursi 2015 [7]	20,990 colorectal cancer patients	82,054 controls	OR	1.16	1.08–1.24	NA	SCH was associated with an increased risk of colorectal cancer.
Fighera 2015 [61]	NA	NA	OR	2.57	1.41–4.70	NA	Risk of thyroid carcinoma increased with increasing TSH levels above 1.64mIU/L.
Tseng 2015 [63]	113,905	1841	RR	1.51	1.06–2.15	NA	SCH was associated with an increased risk of cancer mortality.
Pinter 2017 [62]	548	69	HR	2.1	1.3–3.3	NA	Higher free thyroxine levels (i.e. > 1.66 vs. < 1.66 ng/dl) was associated with a higher overall survival of patients with HCC.

Abbreviations: *n* number of individuals, *CI* confidence interval, *TPOAb* anti- thyroperoxidase antibodies, *NA* not available, *TSH* thyroid stimulating hormone, *HR* hazard ratio, *OR* odds ratio, *RH* relative hazard, *RR* relative risk ratio, *HCC* hepatocellular carcinoma

### Quality assessment

Overall, the cohort studies had good quality (Table 3). The study by Fighera et al. [61] had high risk of bias because the exposed and non-exposed groups did not come from the same source population [61]. Also, it was not clear if SCH was identified prior to the cancer outcome [61]. Two studies did not fully adjust for confounder factors present in the exposed and non-exposed groups [16, 62]. Razvi et al. [16] assessed cancer mortality as a secondary outcome and the variables that were adjusted in the analyses were focused for cardiovascular outcomes. Finally, Pinter et al. [62] assessed the association between thyroid dysfunction and overall survival among patients with hepatocellular carcinoma. Unfortunately, the data collected for these patients lacked information on a number of potential confounders related to patient characteristics, including comorbidities.

**Table 3:** Quality assessment of cohort studies using the Cochrane Tool to assess the risk of bias

Author, year	Selection of exposed and non-exposed from same population?	Can we be confident in the assessment of exposure?	Outcome of interest was not present at start of the study?	The study matched exposed and non-exposed for all the variables associated with the outcome of interest or did the statistical analysis adjust for these prognostic variables?	Can we be confident in the assessment of absence and presence of prognostic factors?	Can we be confident in the assessment of outcome?	Was the follow-up of cohorts adequate?	Were con-interventions similar between groups?
Kuijpens, 2005 [8]	DY	DY	DY	DY	DY	DY	PN	DY
Hellevik, 2009 [15]	DY	DY	DY	PY	DY	DY	PY	PY
Razvi, 2012 [16]	PY	DY	DY	PN	DY	DY	DY	PY
Waring, 2012 [64]	DY	DY	DY	PY	PY	DY	DY	PY
Tseng, 2015 [63]	PY	DY	DY	PY	DY	DY	DY	PY
Fighera, 2015 [61]	DN	DY	PN	PN/PY	DY	DY	PY	PY
Pinter, 2017 [62]	DY	DY	DY	PN	DY	DY	PY	PY

Abbreviations: *DY* definitely yes, low risk of bias, *PY* probably yes, *PN* probably no, *DN* definitely no, high risk of bias

Case control-studies had very good quality overall (Table 4). The identification of cases involved record linkage only to a primary care database in the study by Boursi et al. [7] without independent validation.

**Table 4:** Quality assessment of case-control studies using the Newcastle –Ottawa quality assessment scale

Author, year	Selection				Comparability of cases and controls^e^	Exposure		
	Case definition adequate^a^	Representativ-eness of the cases^b^	Selection of controls^c^	Definition of controls^d^		Ascertainment of exposure^f^	Same method of ascertainment for cases and controls^g^	Non-response rate^h^
Mondul, 2012 [60]	★	★	★	★	★★	★	★	★
Boursi, 2015 [7]	B	★	★	★	★★	★	★	★

^a^:★ = Requires some independent validation (e.g. > 1 person/record/time/process to extract information, or reference to primary record source such as X-rays or medical/hospital records; B = Record linkage (e.g. ICD codes in database) or self-report with no reference to primary record; C = No description

^b^:★All eligible cases with outcome of interest over a defined period of time, all cases in a defined catchment area, all cases in a defined hospital or clinic, group of hospitals, health maintenance organization, or an appropriate sample of those cases (e.g. random sample); B = Not satisfying requirements in part (★), or not stated

^c^:★Community controls (i.e. same community as cases and would be cases if had outcome; B = Hospital controls, within same community as cases (i.e. not another city) but derived from a hospitalized population; C = No description

^d^:★ If cases are first occurrence of outcome, then it must explicitly state that controls have no history of this outcome. If cases have new (not necessarily first) occurrence of outcome, then controls with previous occurrences of outcome of interest should not be excluded; B = No mention of history of outcome

^e^: A maximum of 2 stars can be allotted in this category: either cases and controls must be matched in the design and/or confounders must be adjusted for in the analysis. Statements of no differences between groups or that differences were not statistically significant are not sufficient for establishing comparability. Note: If the odds ratio for the exposure of interest is adjusted for the confounders listed, then the groups will be considered to be comparable on each variable used in the adjustment. Age = ★, other controlled factors = ★

^f^:★ = secure record (e.g. surgical records) or structured interview where blind to case/control status; C = interview not blinded to case/control status; D = written self-report or medical record only; E = no description

^g^:★ = yes; B = no.

^h^:★ = same rate for both groups; B = non-respondents described; C = rate different and no designation

### Cancer incidence

The risk of cancer incidence varied depending on the type of cancer studied. Hellevik et al. [15] found no association between hypothyroidism (i.e. inclusion of SCH and overt hypothyroidism) and overall cancer risk (patients with TSH > 3.5 mIU/L, risk of all cancers adjusted hazards ratio [HR]: 0.96; 95% CI: 0.82–1.12). In this study, individual cancer risks for the more common cancers were also assessed (i.e., lung, colon, prostate, and breast cancer). There were no observed increased risks of lung (adjusted HR: 0.87; 95% CI: 0.43–1.74), colon (adjusted HR: 0.95; 95% CI: 0.60–1.50), prostate (adjusted HR: 0.86; 95% CI: 0.55–1.35), and breast cancer (adjusted HR: 0.85; 95% CI: 0.57–1.25) among patients with a TSH level of > 3.5 mIU/L relative to patients with a TSH level within normal range (TSH: 0.5 to 1.4mIU/L). In contrast, Boursi et al. [7] reported a modest increased risk of colorectal cancer in patients with untreated hypothyroidism and SCH without thyroid hormone replacement compared to euthyroid patients (adjusted odds ratio [OR]: 1.16; 95% CI: 1.08–1.24). This study also assessed the effects of levothyroxine treatment for SCH compared to euthyroid patients and found a protective effect of thyroid hormone replacement against colorectal cancer (adjusted OR: 0.92; 95% CI: 0.86–0.98).

Two studies assessed the association between thyroid dysfunction and breast cancer risk. Kuijpens et al. [8] demonstrated that low levels of free T4 (fT4) are an independent risk factor for the development of breast cancer in peri- and post-menopausal women (risk of cancer in patients with low fT4 levels (≤10th percentile which is equivalent to ≤12.5 pmol/L, OR: 2.3; 95% CI: 1.2–4.6). As mentioned above, Hellevik et al. [15] found no increased risk of breast cancer in patients with hypothyroidism.

Only one study assessed the risk of prostate cancer associated with thyroid function. Mondul et al. [60] reported that hypothyroid men (i.e. inclusion of men with SCH and overt hypothyroidism) had a significantly reduced risk of overall prostate cancer compared to euthyroid men (adjusted OR: 0.71; 95% CI: 0.47–1.06 for TSH ≥ 2.2mIU/L). This result conflicted with that found by Hellevik et al. [15] whereby there was no association between the risk of prostate cancer and SCH or hypothyroidism. Finally, Fighera et al. [61] assessed the risk of thyroid cancer associated with thyroid function and reported an association between serum levels of TSH of > 1.64mIU/L and thyroid malignancy (OR: 2.57; 95% CI: 1.41–4.70).

### Cancer mortality

Four articles reported cancer-related mortality outcomes [16, 62–64]. Pinter et al. [62], assessed overall survival for 667 patients with hepatocellular carcinoma and found that patients with SCH had a slightly decreased survival time compared to euthyroid patients (median overall survival in patients with SCH: 6.1 months; 95% confidence interval [CI]: 0–13.5 versus 10.8 months; 95% CI: 6.5–15.2). Tseng et al. [63], performed a prospective cohort study of 115,746 patients followed in Taiwan with 10 years of follow-up and reported that SCH was independently associated with increased risk of cancer mortality (adjusted relative risk [RR]: 1.51; 95% CI: 1.06–2.15). Their study showed that the risk of cancer mortality among patients with SCH compared to euthyroid patients was more pronounced in bone, skin, and breast cancer. In a sub-analysis from the same study [63] restricted to patients with SCH defined as having TSH levels between 5 and 9.9 mIU/L, the association between SCH and increased cancer-related mortality remained significant (adjusted RR: 1.61; 95% CI: 1.12–2.31). Razvi et al. [16] conducted a retrospective cohort study using the General Practice Research Database (GPRD; since renamed the Clinical Practice Research Datalink), which is representative of patients followed in primary care in the United Kingdom. They determined that patients with SCH between the ages of 40 and 70 years treated with levothyroxine (*n* = 1634) had lower cancer-related mortality compared to untreated patients with SCH (*n* = 1459) (adjusted hazard ratio [HR]: 0.59; 95% CI: 0.21–0.88). Similarly, Waring et al. [64] conducted a prospective study using the Osteoporotic Fractures in Men (MrOS) study cohort which is comprised of 1587 men age ≥ 65 years with baseline thyroid function testing. This study assessed the association between thyroid function, including SCH and the risk of mortality over a mean follow-up of 8.3 years. The results of this study found no association between SCH and the risk of cancer-related mortality compared with euthyroid men (relative hazard: 0.88; 95% CI: 0.44–1.74) (Table 2).

### Anti-thyroperoxidase antibodies

Only one study addressed the association between TPOAb and the risk of cancer, specifically breast cancer risk. Kuijpens et al. [8] found that TPOAb were more prevalent in women with a previous or current diagnosis of breast cancer (OR: 3.0; 95% CI: 1.41–6.46). However, the presence of TPOAb was not associated with the development of incident in situ breast cancer during follow-up (OR: 1.1; 95% CI: 0.4–2.7) (Table 2).

## Discussion

This systematic review assessed the association between SCH and cancer incidence and cancer mortality. We found that SCH was found to be associated with an increased risk of cancer incidence specifically for colorectal and thyroid cancer and cancer-related mortality. A possible protective effect from untreated hypothyroidism against prostate cancer was also found. However, further studies are required to confirm these associations. Despite that thyroid hormone was found to have a role in breast cell proliferation, there is inconsistent evidence that thyroid function affects the risk of breast cancer. One study found no association between thyroid function and breast cancer risk [15]. Only one study showed that low levels of free T4 were an independent risk factor for breast cancer in peri- and post-menopausal women [8]. Few studies assessed the association between the presence of TPOAb and breast cancer risk with controversial results. Therefore, there is insufficient evidence to suggest whether thyroid function or TPOAb levels are associated with breast cancer risk.

Some studies have elucidated potential mechanisms in which thyroid hormone abnormalities can increase the risk of developing certain cancers [9, 65–67]. In breast cancer, it has been hypothesized that an alteration in the iodine metabolism in breast tissues could have a role in its pathogenesis since the sodium-iodine symporter is also present in breast tissue [68]. In addition, T3 can activate thyroid hormone receptors in the breast inducing cell proliferation and lobular growth in a similar fashion as estrogens [69]. In colorectal and prostate cancer, T4 and T3 stimulate the membrane receptor integrin αvβ3, which activates some molecular pathways such as PI3-K and MAPK/ERK1/ 2, triggering cell proliferation and angiogenesis [65]. Recent animal studies have found that the use of tetraiodothyroacetic acid (tetrac), which is a thyroid hormone analogue that inhibits the activation of the membrane receptor integrin αvβ3 on human cancer xenografts lead to tumor regression and decreased tumor growth [70–73].

Specifically for colorectal cancer, there are two additional nuclear receptors with antagonistic effects involved: TRα1 and TRβ1 [66, 67, 74]. The effect on thyroid hormones on TRα1 results in the stimulation of β-catenin producing cellular proliferation in the colon [74]. Conversely, TRβ1 blocks cellular proliferation when activated by thyroid hormones [67]. As such, the lack of TRβ1 expression is associated with malignant transformation in colon cancer [66]. In regards to thyroid cancer, TSH levels has been shown to be an independent clinical predictor of malignancy in thyroid nodules [75] and it is also implicated in the in vitro expression of growth factors associated with cell proliferation and angiogenesis of thyroid cells. These growth factors include the insulin-like growth factor type 1, epidermal growth factor and the vascular endothelial growth factor [55, 76, 77], which could potentially increase the risk of thyroid cancer.

To our knowledge, this is the first systematic review of the literature that analyzes the effect of SCH specifically on the risk of incident cancer and cancer-related mortality. Previous systematic reviews of the literature and meta-analysis focused exclusively on breast cancer [1, 2, 78], and two of these systematic reviews were focused on studying the effect of overt hypothyroidism on breast cancer [1, 78]. Taking into consideration that overt hypothyroidism is almost always treated, these systematic reviews cannot analyze the isolated effect of the hypothyroidism itself on cancer, which constitutes a source of bias from those studies. In addition, it is difficult to determine whether their hypothyroidism persisted throughout follow-up. Furthermore, all of them included only cross-sectional studies [1, 2, 78], which are temporally ambiguous and may be affected by reverse causality whereby the thyroid dysfunction is caused by the development of cancer. As acknowledged by Kuijpens et al. [8], the presence of TPOAb can be concurrent with breast cancer but their presence does not necessarily imply a risk of developing cancer in the future. This observation supports the hypothesis that the presence of concurrent TPOAb and breast cancer in cross-sectional study designs could be more related to reverse causation.

Two large cohort studies found an association between SCH and overall cancer-related mortality. Tseng et al. [63] performed a cohort study involving 115,746 participants and found a 2.06% cancer death rate among individuals with SCH versus 1.31% among euthyroid individuals (*p* = 0.0051). Consistent with this finding, Razvi et al. [16] performed a cohort study using the GPRD, involving 4735 individuals and found a 41% decreased risk of cancer related mortality among younger individuals aged 40 to 70 years who received treatment for SCH compared to untreated individuals with SCH. There was no statistical significant difference in the risk of cancer related mortality among older individuals aged over 70 years with treated versus untreated SCH. This finding is consistent with the study conducted by Waring et al. [64] which utilized data from the MrOS cohort which consists of men ≥65 years. In this study, 1248 euthyroid men were compared with 89 men with subclinical hypothyroidism and there was no association between subclinical hypothyroidism and cancer mortality. These findings suggest that treatment of SCH or being in a euthyroid state may decrease cancer-related mortality among younger individuals. However, these studies are limited by the lack of adjustment for confounding by indication as patients with known cancer may be less likely to receive treatment for SCH if they have a poorer prognosis compared to a cancer patient who is less ill. Furthermore, the findings by Razvi et al. [16] and Waring et al. [64] were from a sub-analysis and the studies did not adjust for all potential confounders in the association between SCH and cancer-related mortality.

Our study has several strengths. We included cohort studies [8, 15, 16, 61–64] and case-control studies [7, 60], excluding cross-sectional studies to address the issue of reverse causality. This systematic review included studies that assessed the risk of various cancers, including the most common cancers such as colorectal, lung, prostate, and breast, and overall cancer risk. In addition, some of the included studies were large, with sample sizes larger than 20,000 patients [7, 15, 63]. All studies had at least 5 years of follow-up, an important consideration for studies of cancer, and potential reverse causality was addressed by excluding studies whereby the cancer diagnosis was made within 12 months of exposure assessment. The quality analysis demonstrated that the included studies had a low risk of bias. Thus, the results of the included studies provided reliable evidence for the association between thyroid dysfunction and cancer incidence and related mortality.

This systematic review had some limitations. There was a high heterogeneity of the studies, especially in terms of different risk measures and outcomes reported. Consequently, we were unable to perform a meta-analysis. Moreover, some of the studies combined individuals with SCH and overt hypothyroidism [7, 8, 15, 60–62], decreasing the strength to the conclusions on the role of SCH in cancer pathogenesis. Furthermore, the study by Pinter et al. [62] diagnosed SCH on the basis of T4 levels, which is different from the definition of SCH used in the other studies. As such, there was heterogeneity in the definition of SCH between studies. Finally, there may be publication bias whereby studies with null findings are not published.

## Conclusions

In summary, SCH may be associated with an increased risk of colorectal and thyroid cancer. Treatment of SCH may be associated with a decreased risk of cancer-related mortality among younger individuals. Overall, there is a paucity of studies addressing the association between SCH and incident cancer risk and cancer mortality. Given the difference of cancer pathophysiology in various cancers, further studies are needed to assess the association between untreated SCH and the risk of different individual cancers.

## Supplementary information

**Additional file 1.**

## Data Availability

Not applicable.

## Abbreviations

CI: Confidence interval
GPRD: General Practice Research Database
HR: Hazards ratio
MrOS: Osteoporotic Fractures in Men
OR: Odds ratio
RR: Relative risk
RXR: Retinoid X receptor
SCH: Subclinical hypothyroidism
T3: Triiodothyronine
T4: Thyroxine
TPOAb: Anti-thyroperoxidase antibodies
TSH: Thyroid stimulating hormone
