# Supplementary material for: Subclinical hypothyroidism and the risk of cancer incidence and cancer mortality: a systematic review
Source: BMC Endocr Disord. 2020 Jun 9;20:83. doi: 10.1186/s12902-020-00566-9 (PMC7285584; doi:10.1186/s12902-020-00566-9)
Supplement: Supplementary file 1 — Additional file 1. [file 12902_2020_566_MOESM1_ESM.docx]

**Appendix**

**Search strategy using Medline.**

| 1 | TSH/ |
| --- | --- |
| 2 | Thyroid stimulating hormone/ |
| 3 | Thyroxin/ |
| 4 | T4/ |
| 5 | Levothyroxin/ |
| 6 | Synthroid/ |
| 7 | Subclinical hypothyroidism/ |
| 8 | Thyroid hormone/ |
| 9 | Thyroid hormone replacement/ |
| 10 | Thyroid hormone receptor/ |
| 11 | 1 OR 2 OR 3 OR 4 OR 5 OR 6 OR 7 OR 8 OR 9 Or 10 |
| 12 | Cancer pathogenesis/ |
| 13 | Angiogenesis/ |
| 14 | Signaling pathways/ |
| 15 | Cancer growth/ |
| 16 | Tumor/ |
| 17 | Tumor promoting/ |
| 18 | Lung cancer/ |
| 19 | Breast cancer/ |
| 20 | Head and neck cancer/ |
| 21 | Melanoma/ |
| 22 | Renal carcinoma/ |
| 23 | Hepatocellular/ |
| 24 | Uterine cancer/ |
| 25 | Ovarian cancer/ |
| 26 | Testicular tumor/ |
| 27 | Colon cancer/ |
| 28 | Prostate cancer/ |
| 29 | Growth suppressors/ |
| 30 | Tumor cells/ |
| 31 | Mutations/ |
| 32 | Inactivating mutations/ |
| 33 | Cancer risk/ |
| 34 | Cancer mortality/ |
| 35 | Risk reduction/ |
| 36 | 12 OR 12 OR 13 OR 14 OR 15 OR 16 OR 16 OR 18 OR 19 OR 20 OR 21 OR 22 OR 23 OR 24 OR 25 OR 26 OR 27 OR 28 OR 29 OR 30 OR 31 OR 32 OR 33 OR 34 OR 35 |
| 37 | 11 AND 36 |

**Pubmed search**

(subclinical[All Fields] AND ("hypothyroidism"[MeSH Terms] OR "hypothyroidism"[All Fields])) AND ("neoplasms"[MeSH Terms] OR "neoplasms"[All Fields] OR "cancer"[All Fields]) AND ("laevothyroxine"[All Fields] OR "thyroxine"[MeSH Terms] OR "thyroxine"[All Fields] OR "levothyroxine"[All Fields]) AND "humans"[MeSH Terms]

(subclinical[All Fields] AND ("hypothyroidism"[MeSH Terms] OR "hypothyroidism"[All Fields])) AND (("neoplasms"[MeSH Terms] OR "neoplasms"[All Fields] OR "cancer"[All Fields]) AND ("risk"[MeSH Terms] OR "risk"[All Fields])) AND "humans"[MeSH Terms]-115 articles retrieved
